# Dermatitis Herpetiformis Duhring

**DOI:** 10.31662/jmaj.2024-0154

**Published:** 2024-09-20

**Authors:** Arisa Kimura, Yasuhito Hamaguchi, Takashi Matsushita

**Affiliations:** 1 Department of Dermatology, School of Medicine, Institute of Medical, Pharmaceutical, and Health Sciences, Kanazawa University, Kanazawa, Japan

**Keywords:** Dermatitis herpetiformis Duhring, IgA, diaminodiphenyl sulfone

A 27-year-old Japanese man experienced severe pruritic erythema for 2 months. He had no symptoms of gluten-sensitive enteropathy. He was initially treated with topical corticosteroids, but his symptoms did not improve. On physical examination, erythematous plaques in an annular-to-continuous circle pattern were observed on the trunk and extremities (Figure 1A). Small vesicles accompanied some of these plaques. Skin biopsy is shown in Figure 2A. Direct immunofluorescence staining revealed IgA deposition in the dermal-epidermal junction papillae (Figure 2B). IgA anti-epidermal transglutaminase antibodies (106.7 AU/mL) and IgA anti-tissue glutaminase antibodies (35.8 AU/mL) were positive by ELISA (reference range, <22 AU/mL). A diagnosis of dermatitis herpetiformis Duhring was made. One month after the administration of diaminodiphenyl sulfone, the patient’s rash abated with pigmentation (Figure 1B). Clinical characteristics of dermatitis herpetiformis Duhring differ between Japanese and Caucasian individuals, with gluten sensitivity less common in Japan ^(1), (2)^.

**Figure 1.**
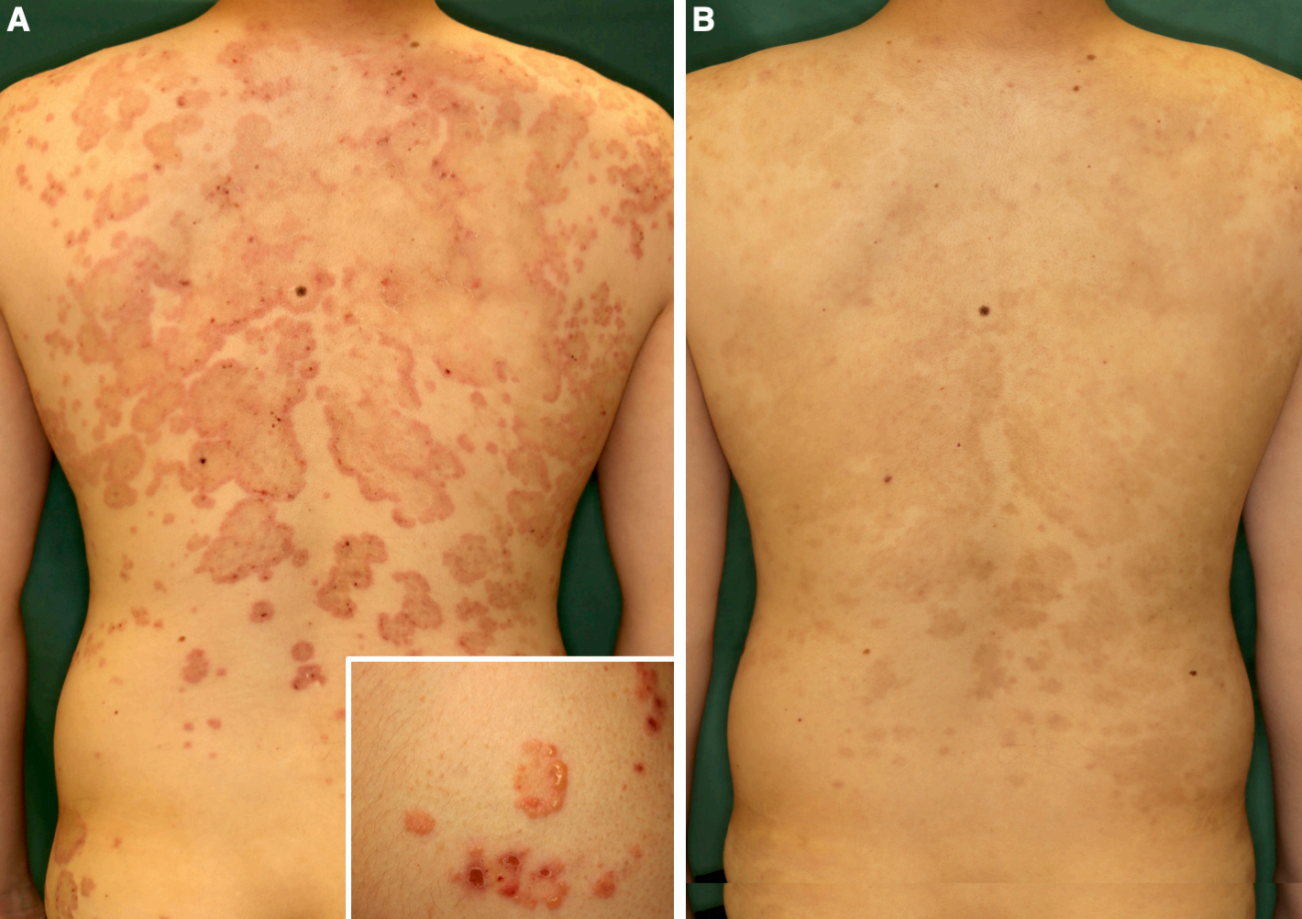
A. Erythematous plaques in an annular-to-continuous circular pattern on the trunk with small vesicles. B. Clinical image in remission status after diaminodiphenyl sulfone treatment.

**Figure 2.**
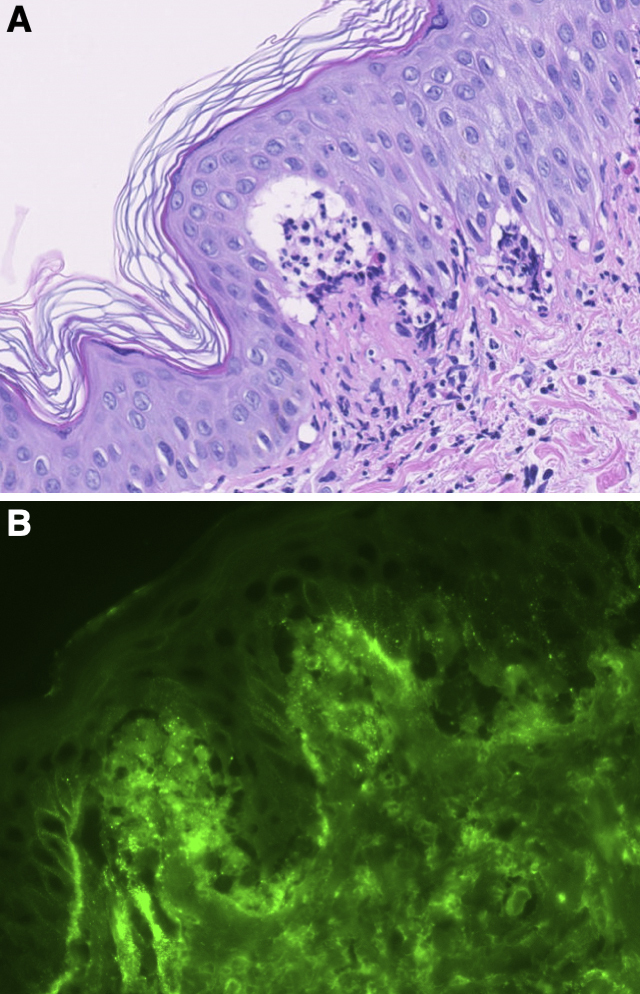
A. Subepidermal blisters and microabscess formation in the papillary layer of the dermis (original magnification, ×200). B. IgA deposition at the dermal-epidermal junction papillae.

## Article Information

### Conflicts of Interest

None

### Acknowledgement

The authors would like to thank Editage (www.editage.com) for the English language editing.

### Author Contributions

Drs. Matsushita and Hamaguchi had full access to all the data in this report and take responsibility for the integrity of the data and the accuracy of the data analysis.

Acquisition of data: Drs. Matsushita and Kimura.

Drafting of the manuscript: Drs. Matsushita, Hamaguchi, and Kimura.

Critical revision of the manuscript and approval of the final version to be submitted: Drs. Matsushita, Hamaguchi, and Kimura.

### Informed Consent

Written informed consent was obtained from the patient for the publication of this case report.
